# The effect of age on the outcomes of cementless mobile bearing unicompartmental knee replacements

**DOI:** 10.1007/s00167-020-06428-0

**Published:** 2021-02-12

**Authors:** Hasan Raza Mohammad, Stephen Mellon, Andrew Judge, Christopher Dodd, David Murray

**Affiliations:** 1 Nuffield Department of Orthopaedics, Rheumatology and Musculoskeletal Sciences, University of Oxford, Nuffield Orthopaedic Centre, Oxford, OX3 7LD UK; 2 Musculoskeletal Research Unit, Bristol Medical School, University of Bristol, Level 1 Learning and Research Building, Southmead Hospital, Westbury-On-Trym, Bristol, BS10 5NB UK; 3 Nuffield Orthopaedic Centre, Windmill Road, Oxford, OX3 7LD UK

**Keywords:** Cementless fixation, Long term outcomes, Unicondylar knee arthroplasty

## Abstract

**Purpose:**

Unicompartmental Knee Replacements (UKR) are being performed in patients with increasing demands and life expectancies with surgical concerns that cemented fixation will not last. Cementless fixation may offer a solution, but the results in different age groups have not been assessed. The effect of age at surgery on the outcomes of cementless UKRs was investigated.

**Methods:**

A prospective cohort of 1000 medial cementless mobile bearing UKR were analysed. Patients were categorised into four age groups (< 55, 55 to < 65, 65 to < 75 and ≥ 75 years). Implant survival was assessed using endpoints reoperation, revision and major revision requiring revision knee replacement components. Functional outcomes were assessed.

**Results:**

10 year cumulative revision rate for the < 55, 55 to < 65, 65 to < 75 and ≥ 75 groups were 2.1% (CI 0.6–6.1), 1.8% (CI 0.6–5.3), 3.2% (CI 1.5–6.5) and 4.1% (1.7–9.6) with no differences between groups (*p* = 0.52). Two of the 22 revisions were considered major. The 10 year cumulative reoperation rates were 4.5% (CI 2.0–10.0), 3.0% (CI 1.3–6.5), 3.8% (CI 2.0–7.1) and 4.1% (CI 1.7–9.6) with no differences between groups (*p* = 0.81). The 10 year median Oxford Knee Scores were 42.5, 46.5, 45 and 42.5, respectively. The 10 year median Objective American Knee Society Scores were 95 for all age groups.

**Conclusion:**

The cementless mobile bearing UKR has low reoperation and revision rates and similar functional outcomes in all age groups. Cementless UKR should be used in all age groups and age should not be considered a contraindication.

**Level of evidence:**

III.

## Introduction

The two main established treatments for end stage medial compartment osteoarthritis and necrosis of the medial condyle are total knee replacement (TKR) and unicompartmental knee replacement (UKR) [29]. UKR offers several advantages over TKR but has higher revision rates in the joint registries [4, 20, 40].

The number of knee replacements is rapidly increasing with a greater proportion of younger patients needing surgery. The under 65 group will form the majority of cases by 2030 [19]. Unfortunately younger age groups have several times higher revision rates [22, 34]. There is surgical concern for both the youngest and oldest subgroups of the population undergoing cementless fixation, particularly for aseptic loosening-related revisions. Younger patients generally have higher levels of activity resulting in greater and more frequent loads being applied to the bone-prosthesis interface [13]. Older patients are more likely to have poor quality bone in which press fit implants are likely to be less reliable [6, 24].

The most commonly used UKR is the Phase 3 Oxford UKR (Biomet, Swindon, United Kingdom) which is designed to be implanted through a minimally invasive approach [29]. The cementless Phase 3 Oxford UKR was introduced in 2004 and has a coating of calcium hydroxyapatite and porous plasma sprayed titanium on its lower (bone-contacting) surface [7]. Cohort studies and randomised controlled trials (RCTs) have demonstrated a reduced incidence of radiolucencies and similar clinical and functional outcomes compared to the cemented Oxford UKR [12, 32]. However, the effect of age on the long term outcomes of the cementless Oxford UKR have not been studied.

This aim of this study is to analyse the effect of age at surgery on the mid to long term clinical outcomes of 1000 cementless UKR. The null hypothesis was that age has no effect on the outcomes of the cementless UKR.

## Materials and methods

Between June 2004 and October 2017, 1000 consecutive medial cementless Oxford UKRs were performed in 870 patients through a minimally invasive approach by two surgeons involved in the design of the implant using the recommended clinical indications [7]. The indications were based on patho-anatomy with the indications being anteromedial osteoarthritis (AMOA) and medial avascular necrosis. Appropriate AMOA cases were those with medial bone-on-bone arthritis, a functionally intact anterior cruciate ligament and full thickness cartilage in the lateral compartment. Age, body mass index (BMI), limb alignment, range of motion and patellofemoral joint arthritis were not considered contra-indications to the procedure.

Age groups were categorised a priori as per the NJR, the Australian Joint Registry and the New Zealand Joint Registry [3, 29, 38]. These groups were patients < 55, 55 to < 65, 65 to < 75, and ≥ 75 years at the time of primary surgery.

Patients were prospectively recruited and assessed preoperatively and at 1-, 2-, 5- and 10 years post operatively by research physiotherapists independent of the surgical teams taking care of the patients. 46 knees were lost from patients dying during the study period; 1 in the under 55 group, 6 in the 55 to < 65 years group, 16 in the 65 to < 75 years group and 23 in 75 plus group. No death was related to the primary operation. Also during the study 44 knees withdrew from regular follow up; 28 knees from patients with poor health, 6 knees from patients going abroad and 10 knees from patients requesting to leave the study. The revision status at the end of the study was known for all patients who died and from all with poor health. Furthermore, none of the patients who were withdrawn from the study were reported by the National Joint Registry of England, Wales, Northern Ireland and Isle of Man (NJR) as having had a revision.

For the survival analysis failure was defined as revision, major revision and reoperation. Revision was defined as the removal, addition or replacement of any implant component as per the joint registries [3, 29, 38]. This, therefore, includes conversions to total knee replacement, patellofemoral replacement and lateral UKR. Major revision was defined as operations requiring the use of TKR with stems, wedges or constraint, which are typically used for revising TKR. Reoperation was defined as any further surgical intervention to the knee and included manipulations under anaesthesia, arthroscopies, fracture fixation and all revisions. The advantage of this outcome is the detection of further operations which are not recorded by the joint registries and which from a patient’s point of view are in many ways similar to a revision.

Patient reported outcome measures (PROMs) were assessed at follow up timepoints using the following metrics; Oxford Knee Score (OKS), American Knee Society Objective Score (AKSS-O), American Knee Society Functional Score (AKSS-F) and the Tegner Activity Score. The AKSS-O was calculated as previously described [25] without deductions if the post-operative alignment was not neutral, as the Oxford UKR does not aim to achieve neutral alignment like TKR, but aims to restore pre-disease alignment [8]. Additionally the Charnley score, maximum knee flexion and the range of extension were also recorded. Flexion was recorded as positive values with hyperextension recorded as negative values.

Complications or further surgeries were recorded when they occurred or at each follow up appointment. Patients who were unable to attend were contacted by post or telephone to obtain the relevant clinical information. The prospective database is updated in real time by a full-time data manager with data extracted on 15th March 2020.

### Statistical analysis

To assess implant survival and cumulative failure rate for both reoperation and revision endpoints the Kaplan–Meier method was utilised. Differences in implant survival between the age groups were tested using the log rank test.

Continuous variables were described using means, standard deviations (SDs), medians and interquartile ranges (IQRs). Categorical variables were tabulated with absolute frequencies. Continuous PROMs data were not normally distributed, and therefore, appropriate nonparametric tests were utilised. To analyse differences in PROMs between the different age groups the Kruskall Wallis test was used.

Maximum extension and flexion data were normally distributed and was, therefore, compared between age groups using the one way analysis of variance. The Charnley score was compared between age groups using the Chi squared proportional test.

Statistical analyses were all performed in Stata version 14 (STATA Corp, TX). *p* values of < 0.05 were considered significant with and 95% confidence intervals (CIs) are reported where appropriate.

## Results

Of the 1000 knees, 989 knees had a diagnosis of anteromedial osteoarthritis and 11 had spontaneous osteonecrosis of the knee. From the 1000 UKRs, 260 were bilateral and of these 4 were simultaneous. 54% of the cohort were male knees, the mean age at surgery was 66.2 years (SD 10.0) and mean BMI was 29.1 (SD 5.0). All patients satisfied the recommended indications [7]. The mean follow-up (*n* = 1000) was 6.5 years (SD 2.7) with 662 and 97 knees having minimum follow up 5 years and 10 years, respectively. The numbers in each age group and their follow up are summarised in Table 1.

**Table 1:** Baseline descriptive statistics of the cohort and number of knees available for analysis at 5- and 10 years follow up

	< 55 years	55 to < 65 years	65 to < 75 years	≥ 75 years	*p* value
Number of knees	151	300	353	196	NA
Number of knees with 5 years minimum follow up	111	210	230	111	NA
Number of knees with 10 years minimum follow up	21	27	39	10	NA
Mean age	50.8 (SD 3.4)	60.4 (SD 2.9)	70 (SD 3.0)	80.1 (SD 3.8)	NA
Mean BMI	30.5 (SD 5.4)	29.6 (SD 5.0)	29.2 (SD 4.9)	27.1 (SD 4.0)	0.001
Sex (proportion male)	0.52	0.55	0.52	0.55	0.84
Preop OKS	22.7 (SD 9.3) 22.0 (IQR 13.0)	26.1 (SD 8.4) 27.0 (IQR 12.0)	25.2 (SD 8.0) 26.0 (IQR 11.0)	25.2 (SD 8.4) 25.0 (IQR 11.0)	0.008
Preop Tegner	2.4 (SD 1.2) 2.5 (IQR 1.5)	2.6 (SD 1.2) 3.0 (IQR 1.0)	2.4 (SD 1.1) 2.0 (IQR 1.0)	2.0 (SD 1.0) 2.0 (IQR 2.0)	< 0.001
Preop AKSS-O	57.0 (SD 15.3) 57.0 (IQR 24.0)	61.3 (SD 15.8) 60.0 (IQR 21.0)	60.2 (SD 14.5) 60.5 (IQR 19.0)	61.1 (SD 16.3) 62.5 (SD 22.0)	0.37
Preop AKSS-F	71.8 (SD 16.5) 70.0 (IQR 20.0)	76.3 (SD 16.0) 80.0 (IQR 20.0)	69.6 (SD 16.0) 70.0 (IQR 20.0)	63.7 (SD 16.6) 65.0 (IQR 15.0)	< 0.001

(NA, not applicable). BMI was compared between groups with one way analysis of variance, sex using the chi squared proportional test and preoperative PROMs using the Kruskall Wallis test

The baseline characteristics between the different age groups are also shown in Table 1. The sex proportions, preoperative Tegner scores, AKSS-O and BMI were similar between groups. Although the mean BMI and preoperative Tegner were significantly different, the absolute differences remained small. The preoperative OKS was lower in the < 55 group and the preoperative AKSS-F higher in the 55 to < 65 group.

**Fig. 1:**
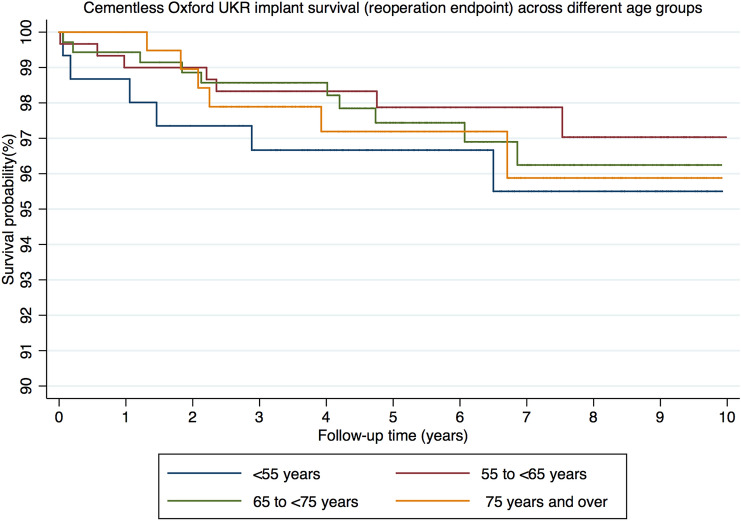
Kaplan–Meier graph of cementless Oxford UKR implant survival (reoperation endpoint) across different age groups

There were 30 reoperations at a mean of 3.0 years (SD 2.7). The details are summarised in Table 2. Using reoperation as an endpoint the 5- and 10 year implant survival of the < 55 group was 96.7% (CI 92.2–98.6) and 95.5% (CI 90.0–98.0), for the 55 to < 65 group was 97.9% (CI 95.3–99.1) and 97.0% (CI 93.5–98.7), for the 65 to < 75 was 97.4% (CI 94.9–98.7) and 96.2% (CI 92.9–98.0) and for the ≥ 75 group was 97.2% (CI 93.3–98.8) and 95.9% (CI 90.4–98.3) (Fig. 1). There were no significant differences in implant survival between groups at both 5- and 10 years (*p* = 0.85 and *p* = 0.81, respectively).

**Table 2:** Details of reoperations and revisions in each age group (^a^major revision)

Age group	Number of reoperations	Number of revisions	Details of reoperations/revisions	Indication for surgery
< 55 years	6	3	2 bearing exchange	2 bearing dislocations
			2 arthroscopies	1 for pain and 1 for knee swelling
			1 open debridement, lavage and bearing exchange	Infection
			1 washout debridement and closure	Wound dehiscence
55 to < 65 years	7	4	1 TKR	Lateral tibial plateau fracture^a^
			1 lateral UKR	Disease progression
			2 arthroscopies	1 for pain and 1 for lateral meniscal tear
			1 arthroscopy and washout	Suspected infection
			1 cemented femoral component revision	Femoral component loosening
			1 tibial component revision	Pain
65 to < 75 years	11	9	3 bearing exchange	Bearing dislocation
			1 DAIR and bearing exchange	Suspected infection
			3 lateral UKRs	Disease progression
			2 TKRs	1 for pain and 1 for disease progression^a^
			1 arthroscopy and arthrotomy	Loose body
			1 aspiration and manipulation under anaesthesia	Pain and intermittent swelling/stiffness
≥ 75 years	6	6	3 bearing exchange	Bearing dislocation
			1 lateral UKR	Tibial avascular necrosis
			1 TKR	Disease progression
			1 patellofemoral replacement	Pain

**Fig. 2:**
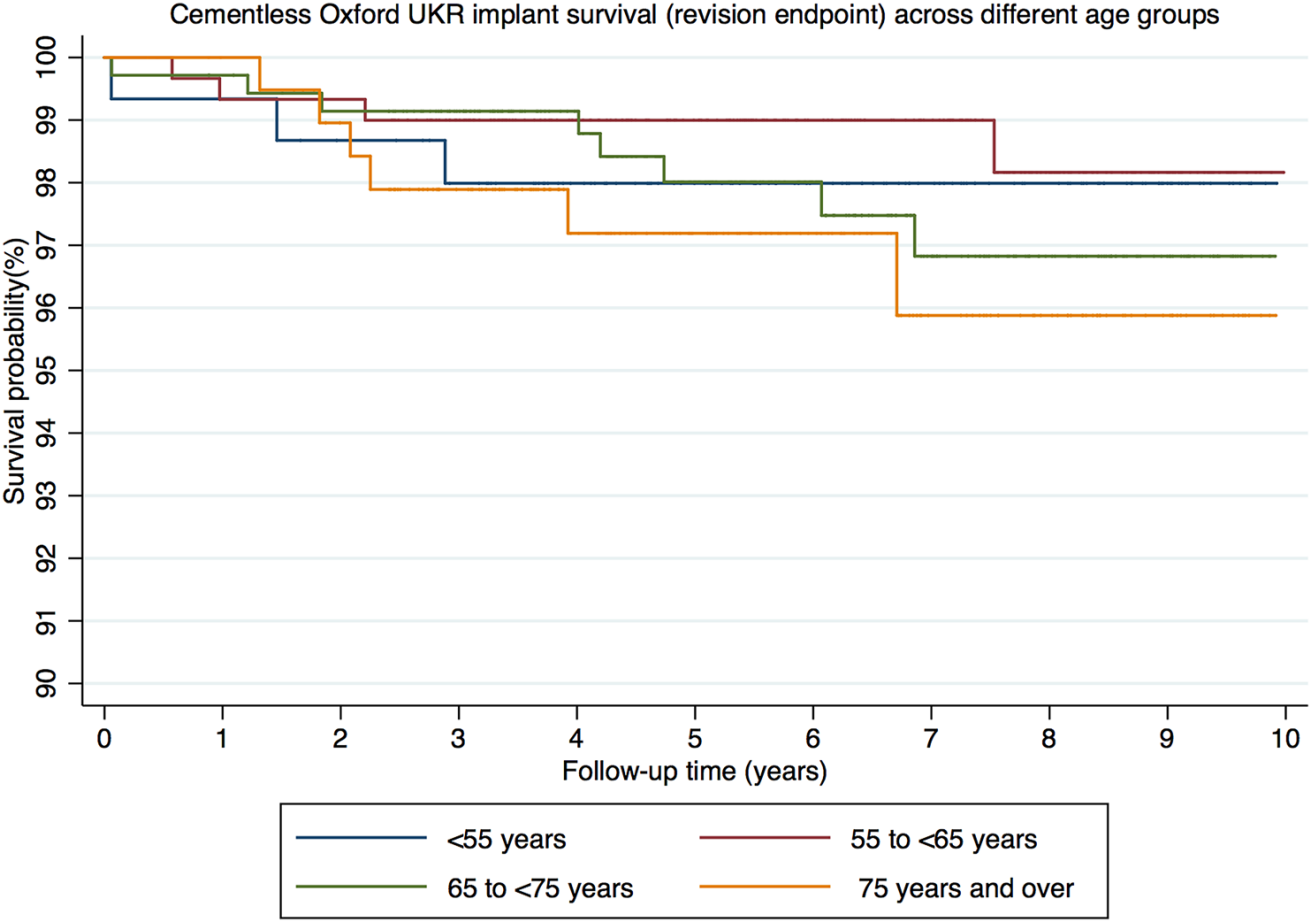
Kaplan–Meier graph of cementless Oxford UKR implant survival (revision endpoint) across different age groups

From the 30 reoperations, 22 met the definition of implant revisions at mean 3.3 years (SD 2.8). Using revision as an endpoint the 5- and 10 year implant survival of the < 55 group was 97.9% (CI 93.9–99.4) and 97.9% (CI 93.9–99.4), for the 55 to < 65 group was 99.0% (CI 96.9–99.7) and 98.2% (CI 94.7–99.4), for the 65 to < 75 was 98.0% (CI 95.6–99.1) and 96.8% (CI 93.5–98.5) and for the ≥ 75 group was 97.2% (CI 93.3–98.8) and 95.9% (CI 90.4–98.3) (Fig. 2). There were no significant differences in implant survival between groups at both 5- and 10 years (p = 0.58 and p = 0.52, respectively). The details of the revisions are summarised in Table 2. There were four revisions to TKRs in the cohort. Two of these were in the 65 to < 75 group, one in the ≥ 75 group and one in the 55 to < 65 group. There were five revisions with lateral UKRs. Three of these were in the 65 to < 75 group, two in the ≥ 75 group and one in the 55 to < 65 group. There was one revision to a patellofemoral replacement, and this was in the ≥ 75 group.

There were two major revisions in the cohort (one in the 55 to < 65 group and the other in the 65 to < 75 group). One knee was converted to a TKR with a stemmed tibial implant following a lateral tibial plateau fracture after a fall and one knee was converted to TKR with tibial stem for lateral disease progression.

**Fig. 3:**
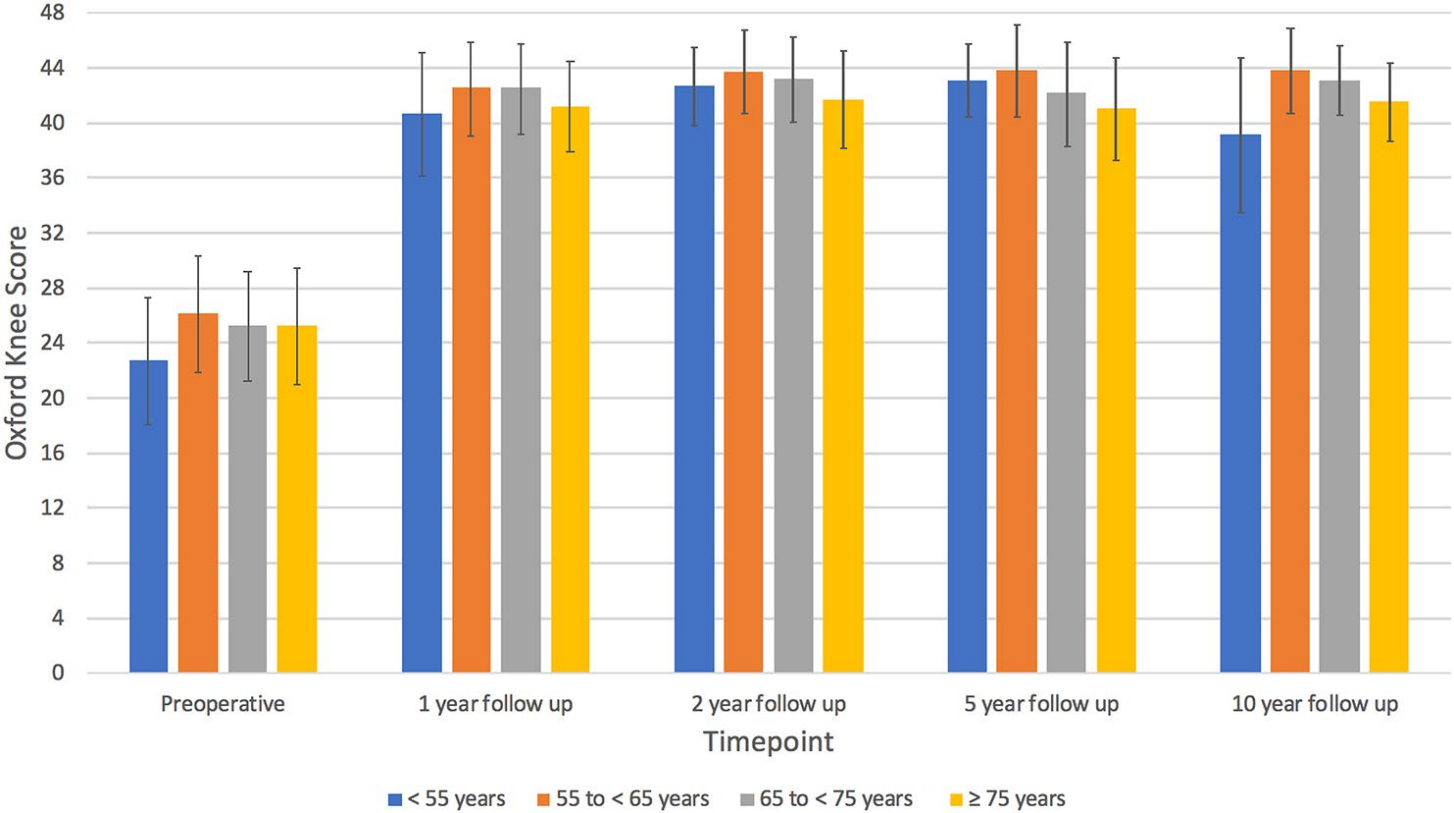
Bar chart of the mean OKS at different time points in the different age groups. Error bars represent the SD

The mean and median post-operative OKS, AKSS-O, AKSS-F and Tegner scores at 1-, 2-, 5- and 10 years improved in all age groups (Table 3) compared to each group’s respective preoperative PROM scores (Table 1). Comparing the post-operative OKS between age groups found that although there were some significant differences, at all-time points any differences between groups were small and of little clinical significance (Table 3). Post operatively and at 10 years follow up the < 55 group had a lower OKS but this group also had the worst preoperative OKS (Fig. 3). With regard to the postoperative AKSS-O, again any significant differences between age groups were small at the different time points assessed. The < 55 group had the lowest preoperative score (Table 3). For the post-operative AKSS-F, at one, two and five years the < 55 and 55 to < 65 group scored highest although these groups had higher preoperative scores. At 10 years there were no significant differences between groups.

**Table 3:** Post-operative Outcomes in the different age groups at different time points

	Age group				Significance
	< 55 years	55 to < 65 years	65 to < 75 years	≥75 years	*p* value
1 year					
1 year OKS	40.6 (SD 7.4) 43.0 (IQR 9.0)	42.5 (SD 6.8) 45.0 (IQR 6.0)	42.5 (SD 6.6) 45.0 (IQR 6.0)	41.2 (SD 6.6) 44.0 (IQR 8.0)	< 0.001
1 year Tegner	3.4 (SD 1.1) 3.0 (IQR 1.0)	3.4 (SD 1.2) 3.0 (IQR 1.0)	3.0 (SD 1.1) 3.0 (IQR 2.0)	2.5 (SD 1.1) 2.5 (IQR 1.0)	< 0.001
1 year AKSS-O	88.7 (SD 14.3) 95.0 (IQR 7.0)	92.6 (SD 10.8) 95.0 (IQR 7.0)	92.2 (SD 11.0) 95.0 (IQR 7.0)	91.6 (SD 10.0) 94.0 (IQR 8.0)	0.03
1 year AKSS-F	89.8 (SD 13.4) 100.0 (IQR 20.0)	89.8 (SD 14.7) 100.0 (IQR 20.0)	85.3 (SD 15.6) 90.0 (IQR 30.0)	76.4 (SD 15.3) 80.0 (IQR 10.0)	< 0.001
1 year Charnley score	29.1% A	24.3% A	19.4% A	23.7% A	0.36
	48.7% B	49.0% B	48.0% B	43.6% B	
	22.2% C	26.8% C	32.6% C	32.7% C	
1 year max flexion (degrees)	129.5 (SD 10.5) 131.5 (IQR 16)	127.8 (SD 11.4) 130.0 (IQR 13)	126.7 (SD 10.5) 128.0 (IQR 12.0)	125.9 (SD 9.9) 125.0 (IQR 12.0)	0.07
1 year max extension	2.5 (SD 3.4) 0.0 (IQR 5)	2.8 (SD 4.1) 2.0 (IQR 5.0)	3.5 (SD 4.2) 3.0 (IQR 5.0)	4.1 (SD 4.6) 3.0 (IQR 7.0)	0.02
2 year					
2 year OKS	42.7 (SD 5.7) 44.0 (IQR 7.0)	43.7 (SD 6.1) 46.0 (IQR 4.0)	43.2 (SD 6.2) 46.0 (IQR 6.0)	41.7 (SD 7.0) 45.0 (IQR 9.0)	0.002
2 year Tegner	3.8 (SD 1.3) 4.0 (IQR 2.0)	3.5 (SD 1.1) 3.0 (IQR 1.0)	3.1 (SD 1.3) 3.0 (IQR 2.0)	2.5 (SD 1.2) 2.0 (IQR 1.0)	< 0.001
2 year AKSS-O	93.2 (SD 8.3) 95.0 (IQR 5.0)	93.5 (SD 10.1) 95.0 (IQR 7.0)	93.4 (SD 10.3) 97.0 (IQR 7.0)	92.6 (SD 10.5) 95.0 (IQR 5.0)	0.24
2 year AKSS-F	90.3 (SD 13.9) 100.0 (IQR 20.0)	89.9 (SD 14.6) 100.0 (IQR 20.0)	85.2 (SD 16.0) 90.0 (IQR 20.0)	76.5 (SD 16.1) 80.0 (IQR 25.0)	0.001
2 year Charnley score	25.8% A	17.9% A	19.5% A	19.9% A	0.15
	44.3% B	53.2% B	45.6% B	43.5% B	
	29.9% C	28.9% C	34.9% C	36.6% C	
2 year max flexion	130.5 (SD 9.1) 130.0 (IQR 12.0)	129.7 (SD 10.4) 131.0 (IQR 12.0)	127.6 (SD 10.2) 128.0 (IQR 13.0)	125.3 (SD 9.0) 125.0 (IQR 10.0)	0.001
2 year max extension	2.0 (SD 3.5) 0.0 (IQR 5.0)	2.5 (SD 3.8) 2.0 (IQR 5.0)	3.2 (SD 4.1) 2.0 (IQR 5.0)	3.4 (SD 4.3) 3.0 (IQR 5.0)	0.05
5 year					
5 year OKS	43.1 (SD 5.4) 45.0 (IQR 6.0)	43.8 (SD 6.6) 46.5 (IQR 5.0)	42.1 (SD 7.5) 45.0 (IQR 7.0)	41.0 (SD 7.4) 44.0 (IQR 9.0)	< 0.001
5 year Tegner	3.6 (SD 1.5) 3.5 (IQR 1.0)	3.4 (SD 1.3) 3.0 (IQR 1.0)	2.9 (SD 1.3) 3.0 (IQR 1.0)	2.3 (SD 1.1) 2.0 (IQR 1.0)	< 0.001
5 year AKSS-O	94.7 (SD 6.5) 95.0 (IQR 7.0)	94.5 (SD 10.3) 98.0 (IQR 5.0)	92.6 (SD 9.4) 95.0 (IQR 8.0)	91.9 (SD 10.1) 95.0 (IQR 8.0)	0.002
5 year AKSS-F	87.3 (SD 18.4) 100.0 (IQR 20.0)	89.3 (SD 14.0) 100.0 (IQR 20.0)	81.9 (SD 17.4) 80.0 (IQR 30.0)	73.7 (SD 19.8) 75.0 (IQR 30.0)	0.001
5 year Charnley score	22.8% A	8.9% A	14.9% A	12.8% A	0.02
	39.2% B	54.1% B	43.7% B	34.9% B	
	38.0% C	36.9% C	41.4% C	52.3% C	
5 year max flexion	130.1 (SD 8.7) 131.0 (IQR 11.0)	130.7 (SD 8.7) 130.0 (IQR 11.0)	126.7 (SD 10.1) 126.0 (IQR 14.0)	123.5 (SD 10.0) 123.0 (IQR 14.0)	< 0.001
5 year max extension	1.6 (SD 3.4) 0.0 (IQR 4.0)	2.5 (SD 3.6) 2.0 (IQR 5.0)	2.3 (SD 4.4) 1.0 (IQR 5.0)	2.0 (SD 5.1) 0.0 (IQR 5.0)	0.47
10 year					
10 year OKS	39.1 (SD 11.2) 42.5 (IQR 10.0)	43.8 (SD 6.1) 46.5 (IQR 6.0)	43.1 (SD 5.0) 45.0 (IQR 7.0)	41.5 (SD 5.7) 42.5 (IQR 10.5)	0.36
10 year Tegner	3.1 (SD 1.7) 3.0 (IQR 1.5)	3.1 (SD 1.2) 3.0 (IQR 1.0)	2.6 (SD 0.9) 3.0 (IQR 1.0)	2.0 (SD 1.0) 2.0 (IQR 2.0)	0.16
10 year AKSS-O	90.2 (SD 13.1) 95.0 (IQR 6.0)	87.9 (SD 17.4) 95.0 (IQR 7.0)	92.5 (SD 7.7) 95.0 (IQR 10.0)	96.0 (SD 2.9) 95.5 (IQR 4.0)	0.95
10 year AKSS-F	78.1 (SD 23.2) 80.0 (IQR 35.0)	81.8 (SD 13.7) 80.0 (IQR 30.0)	80.8 (SD 17.9) 80.0 (IQR 30.0)	69.0 (SD 10.2) 65.0 (IQR 20.0)	0.40
10 year Charnley score	6.7% A	4.6% A	6.5% A	25.0% A	0.04
	33.3% B	13.6% B	41.9% B	25.0% B	
	60.0% C	81.8% C	51.6% C	50.0% C	
10 year max flexion	131.1 (SD 8.6) 131.0 (IQR 4.0)	129.8 (SD 10.7) 133.0 (IQR 14.0)	126.6 (SD 8.7) 125.0 (IQR 10.0)	131.3 (SD 12.1) 129.0 (IQR 15.5)	0.52
10 year max extension	2.8 (SD 3.2) 2.0 (IQR 5.0)	3.2 (SD 4.5) 3.0 (IQR 5.0)	2.7 (SD 5.4) 2.0 (IQR 8.0)	3.3 (SD 3.8) 3.0 (IQR 6.5)	0.99

PROMs were compared with the Kruskall Wallis test, Charnley scores with the Chi squared proportional test and flexion/extension with one way anova test

High mean flexion angles in a range of 125 to 130 degrees were achieved in all age groups at all time points. The mean extension angles were between 2 to 4 degrees at all time points for all age groups.

At 1-, 2- and 5 years there was a tendency for a greater proportion of patients with Charnley C scores in the older age groups. However, at 10 years follow up this was not observed, perhaps because in all groups the co-morbidities increased with age but in the oldest group a higher proportion of those with greater co-morbidities would have died. Subgroup analyses comparing the OKS of knees with Charnley scores of A and B compared to C in each age group are presented in Table 4. There were insufficient numbers to perform this analysis at 10 years follow up. In all age groups at all time points the Charnley groups A and B scored higher, by about two or three OKS points, than those of group C. Other than this the differences between ages in the various subgroups were small and inconsistent.

**Table 4:** The OKS in different Charnley groups within each age group

Age group	Charnley group	1 year	2 year	5 year
< 55 years	A and B	41.4 (SD 6.8) 44.0 (IQR 7.0)	43.4 (SD 5.9) 45.5 (IQR 4.5)	43.3 (SD 5.2) 45.0 (IQR 5.0)
	C	38.5 (SD 8.0) 40.0 (IQR 13.0)	41.3 (SD 4.9) 41.0 (IQR 7.0)	43.0 (SD 5.8) 44.5 (IQR 5.0)
55 to < 65 years	A and B	43.1 (SD 6.1) 45.0 (IQR 5.0)	44.7 (SD 5.0) 46.0 (IQR 4.0)	44.8 (SD 4.9) 47.0 (IQR 4.0)
	C	40.8 (SD 8.5) 44.0 (IQR 8.0)	41.4 (SD 7.5) 44.0 (IQR 7.0)	42.7 (SD 7.8) 46.0 (IQR 5.0)
65 to < 75 years	A and B	43.3 (SD 6.0) 45.5 (IQR 5.0)	44.4 (SD 5.3) 46.0 (IQR 4.0)	44.0 (SD 5.8) 46.0 (IQR 4.0)
	C	40.8 (SD 7.7) 44 (IQR 9.0)	41.3 (SD 7.4) 44.0 (IQR 8.0)	40.0 (SD 8.4) 42.0 (IQR 11.0)
≥ 75 years	A and B	41.9 (SD 6.2) 44.0 (IQR 7.0)	42.5 (SD 6.4) 45.0 (IQR 8.0)	42.8 (SD 6.5) 45.0 (IQR 7.0)
	C	40.0 (SD 7.0) 41.0 (IQR 8.0)	40.3 (SD 7.8) 43.0 (IQR 9.0)	39.7 (SD 8.1) 43.0 (IQR 8.0)

## Discussion

To the best of the author’s knowledge this is the first study to investigate the effect of age at surgery on the mid to long term outcomes of a cementless unicompartmental knee replacement. The most important finding is that excellent long term implant outcomes were achieved for all age groups. In all age groups the survival for both revision and reoperation endpoints, exceeded 95% at 10 years. Additionally there were no significant differences in implant survival between groups. This suggests that cementless UKR should be used in all age groups and age should not be a contraindication. The results of the study are different to the reports from the registries which suggest that revision rates increase dramatically with decreasing age [3, 29, 38].

In the NJR the 10 year cumulative revision rate for UKR in patients < 55 yr is about 17%, yet in this study it was 2.1% [29]. Unfortunately the NJR does not stratify age analysis by fixation type, but the type of fixation would not account for such a large difference. It is more likely to relate to the indications for primary surgery [14]. In the NJR most surgeons do very small numbers of UKR, with the commonest being one or two per year [21]. Given that the recommended indications for Oxford UKR are satisfied in about 50% of knee replacements these surgeons are not adhering to the recommended indications [39]. More importantly they seem to be using UKR for younger patients [29] with early disease without bone-on-bone arthritis in whom they are reluctant to perform TKR surgery [14]. Patients with partial thickness loss have been shown to have poor outcomes with high revision rates [9, 16, 30]. Indeed Kennedy et al. [14] reviewed the pre-operative radiographs of Oxford UKR revisions identified by the NJR and found that in about one third of cases there was not bone-on bone arthritis before the primary procedure.

The main theoretical concern about changing from cemented to cementless fixation is that there might be an increased risk of aseptic loosening particularly in the youngest and oldest age groups [5]. The youngest groups are of concern given their increased activity, as is reflected in this study by their higher Tegner scores. It is expected that patients with higher levels of activity will have an increased probability of implant mechanical failure [13]. However, this study and others in the literature [1, 15, 37] do not suggest this the case for mobile bearing UKR, which probably relates to the design of the implant. The mobile bearing is fully congruent minimising contact stresses and hence wear [36]. Additionally given the bearing is mobile the loads are predominantly compressive which reduces the risk of aseptic loosening from shear forces [31]. The implant also aims to maintain knee kinematics by preserving the ligamentous structures. The youngest group had no revisions for aseptic loosening in this study supporting this concept. Additionally there were no cases of disease progression likely reflecting the healthier cartilage in the lateral compartment for this group.

The oldest age group (≥ 75 years) is also of concern given the generally poorer quality of bone; however, in this study, there were no cases of aseptic loosening in this group. This supports the notion that the Oxford UKR achieves similar clinical outcomes in patients with generally reduced bone mineral density [24]. This is probably partly, because with the Oxford UKR, the bone resection is minimal as 3 mm or 4 mm bearings are usually used so the retained subchondral bone is relatively dense. In addition the patients have varus in the knee because of the arthritis and often had pre-existing varus. This would increase the loading in the medial compartment, helping to preserve bone density and provide secure cementless fixation despite generalised osteoporosis [8].

All age groups had improvements in PROMs post operatively at all timepoints compared to their respective preoperative scores. Although there were some significant differences between age groups these absolute differences were generally small suggesting similar functional outcomes in all age groups. For example the OKS tended to be slightly lower in the < 55 age group than the others. The improvement in OKS was, however, similar as the pre-operative OKS was appreciably lower in this age group, presumably because the surgeons were trying to delay the operation as long possible. The AKSS-F was lower in the > 75 age group particularly at ten years, which is presumably a manifestation of these patients being older and frailer than the younger age groups. The Charnley A and B knees had higher OKS than group C in all age groups. The difference was about two or three OKS points and is probably a manifestation of their systemic problems compromising their knee function. The fit patients in all age groups achieved extremely high median OKS of 45–47 (out of 48).

This is the first study to investigate the effect of age on the long term outcomes of a cementless mobile bearing unicompartmental knee replacement. Several studies have investigated the effect of age on the outcomes of cemented mobile bearing UKR, although these generally only categorised age into two groups (older and younger than 60 years of age) and show conflicting results [11, 17, 18, 33, 35]. However, when the results of this cohort are compared to a previously published similar cemented cohort [13] from the same operating surgeons using the same age group categorisations, the cementless did better in all age groups: In age groups < 55, 55 to < 65, 65 to < 75 and ≥ 75 the 10 years cumulative revision rates in the cemented [13] were 3%, 6%, 6% and 7% and in the cementless were 2%, 2%, 3%, and 4%. Matched studies based on NJR data [26, 27] have shown significantly lower revision rates in cementless than cemented UKR so the improvement seen with cementless in the two cohort studies is probably real. The lower revision rate of the cementless was primarily due to reduced rates of revision for aseptic loosening and pain suggesting that cementless fixation is better than cemented. Furthermore, in both the previously published cemented UKR study [13] and this study there was a similar trend with decreasing revision rates in younger patients suggesting that this also is real. This is presumably because the implant is robust and in younger patients the bone and preserved articular cartilage is of better quality so less likely to fail despite higher activity levels.

The surgeons involved in this study were adhering to the recommended indications described in detail in the methods section and were using UKR for more than half of their primary knee replacements. Most surgeons worldwide would have done TKR in these patients. There is now persuasive evidence that UKR provides better functional outcomes than TKR [40]. This is supported by the high levels of PROMs and range of movement achieved in this study, which are better than that achieved by TKR. The controversy relates to failure rates. To explore this the cumulative rates of re-operation, revision and major revision were determined at ten years. For all age groups the re-operation rate was less than 5% which is less than that of TKR, as these are associated with higher numbers of manipulations under anaesthesia and operations for possible infection [23, 41]. Similarly the rates of major revision requiring stems, wedges or stabilisation are lower for UKR as almost all TKR revisions are major and, in this study, only 0.2% had major primary revisions. The controversy that exists, therefore, still relates to revision, which due the widespread use of registries has become accepted to be any re-operation in which a new implant is inserted. In the < 55 age group the revision rate of cementless UKR of 2% is half of the 5.4% reported by Aujla et al. in their systematic review of TKRs performed in the under 55 s [2]. This is an important finding given the life expectancy of these patients. In contrast the revision rate in those > 75 is higher than TKR [29]. However, as these patients have limited life expectancy the number of excess revisions will be minimal and of less consequence than the higher morbidity and mortality of TKR [20]. In the intermediate ages groups the revision rate of cementless UKR and TKR [29] are similar, so UKR would have advantages over TKR but no disadvantage.

The main strengths of this observational study are that it is a large prospective consecutive series of 1000 cementless Oxford UKRs with the recommended surgical indications and independent follow up. Additionally several outcome measures were assessed pertaining to both implant survival and functional outcomes achieved. This information is not available in the joint registries.

This study does, however, have important limitations. This is a single centre study from the surgeons involved in the design of the Oxford UKR which limits its generalisability. However, if surgeons adhere to the recommended indications for the Oxford UKR their results have been shown to be similar to those of the designer surgeons [10]. Additionally the results pertain to the Oxford UKR and, therefore, may not be generalisable to all types of cementless UKRs, as with the mobile bearing the loads applied to the bone-prosthesis interface are predominantly compressive with minimal shear or tension which is ideal for cementless fixation [31]. The numbers available for analysis at 10 years were limited which is reflected in the confidence intervals but the implant survivals were similar in all age groups. Finally given this is not a randomised controlled trial of different patient age groups undergoing cementless UKR surgery. Therefore there were some differences in the baseline characteristics of the age groups. This included the lower PROMs scores in the < 55 years group which would be expected given surgeons reluctance to operate on young patients unless absolutely necessary. Additionally the younger groups had higher BMI as would be expected given high BMI is an important risk factor for knee arthritis. However, previous research has shown that BMI does not affect the long term outcomes of the Oxford UKR [28].

## Conclusions

The cementless mobile bearing UKR is a safe procedure in all age groups with no significant differences in the mid to long term outcomes including reoperation, revision and functional status. Age should, therefore, not be considered a contraindication to the cementless mobile bearing UKR.
